# Association between parity and dentition status among Japanese women: Japan public health center-based oral health study

**DOI:** 10.1186/1471-2458-13-993

**Published:** 2013-10-22

**Authors:** Masayuki Ueno, Satoko Ohara, Manami Inoue, Shoichiro Tsugane, Yoko Kawaguchi

**Affiliations:** 1 Department of Oral Health Promotion, Graduate School of Medical and Dental Sciences, Tokyo Medical and Dental University, Tokyo, Japan; 2 Department of Comprehensive Oral Health Care, Faculty of Dentistry, Tokyo Medical and Dental University, Tokyo, Japan; 3 AXA Department of Health and Human Security, Graduate School of Medicine, University of Tokyo, Tokyo, Japan; 4 Epidemiology and Prevention Division, Research Center for Cancer Prevention and Screening, National Cancer Center, Tokyo, Japan

**Keywords:** Parity, Dentition status, Dental caries, Periodontal disease, Oral health

## Abstract

**Background:**

Several studies have shown that parity is associated with oral health problems such as tooth loss and dental caries. In Japan, however, no studies have examined the association. The purpose of this study was to determine whether parity is related to dentition status, including the number of teeth present, dental caries and filled teeth, and the posterior occlusion, in a Japanese population by comparing women with men.

**Methods:**

A total of 1,211 subjects, who participated both in the Japan Public Health Center-Based (JPHC) Study Cohort I in 1990 and the dental survey in 2005, were used for the study. Information on parity or number of children was collected from a self-completed questionnaire administered in 1990 for the JPHC Study Cohort I, and health behaviors and clinical dentition status were obtained from the dental survey in 2005. The association between parity or number of children and dentition status was analyzed, by both unadjusted-for and adjusted-for socio-demographic and health behavioral factors, using a generalized linear regression model.

**Results:**

Parity is significantly related to the number of teeth present and n-FTUs (Functional Tooth Units of natural teeth), regardless of socio-demographic and health behavioral factors, in female subjects. The values of these variables had a significantly decreasing trend with the rise of parity: numbers of teeth present (p for trend = 0.046) and n-FTUs (p for trend = 0.026). No relationships between the number of children and dentition status were found in male subjects.

**Conclusion:**

Higher-parity women are more likely to lose teeth, especially posterior occluding relations. These results suggest that measures to narrow the discrepancy by parity should be taken for promoting women’s oral health. Delivery of appropriate information and messages to pregnant women as well as enlightenment of oral health professionals about dental management of pregnant women may be an effective strategy.

## Background

Pregnancy and parturition have a tremendous effect on maternal health. Complications of pregnancy include bleeding, premature rupture of the membranes, puerperal endometritis and anemia [1,2]. In the worst cases they cause serious conditions and death of the mothers [3]. Alarming oral health problems related to maternity have also been reported. There is a commonly used proverb, not only in Japan but in other countries, that a mother loses one tooth every time she gives birth to a child. Several studies show that parity (i.e., the number of children to which a woman gives birth) is associated with oral health conditions such as tooth loss and dental caries [4-6].

In a Danish study [4], the number of teeth present in women was negatively correlated with the number of their children. Women in low social status lost about one additional tooth per child, while those in high social status lost about one additional tooth per two children. In the same study, among identical female twins, the twin with more children had fewer teeth. For male twin pairs, such clear relationships were not found.

The third National Health and Nutritional Examination Survey (NHANES III) demonstrated that parity was related to tooth loss among American women [5]. Among Black and White Non-Hispanic American women in this survey, increased parity was also related to a higher number of untreated decayed surfaces [6]. However, some studies have not found an association between parity and oral health conditions [7,8].

To date, there is a paucity of research on the relationship between parity and oral health status. In Japan, in particular, no studies have been conducted to examine this association. Further, this kind of research will become more difficult in the future due to the declining birthrate in Japan; the birthrate in 2012 was 1.41 [9]. Therefore, the purpose of this study was to determine whether parity is related to dentition status, including the number of teeth present, dental caries, filled teeth and posterior occlusion, in a Japanese population by comparing women with men.

## Methods

### Subjects

In 1990, the Japan Public Health Center-Based (JPHC) Study Cohort I was launched in order to prospectively follow the morbidity and mortality of diseases, such as cancer and cardiovascular diseases, in a large population-based Japanese sample [10]. In 2005, a dental survey was conducted for the sub-cohort of the Yokote health center jurisdiction, Akita Prefecture from the JPHC Study Cohort I. Therefore, subjects in this study were those who had participated both in the JPHC Study Cohort I in 1990 and the dental survey in 2005.

We mailed invitation letters to 15,782 eligible residents, aged 55 to 75 years as of May, 2005, to inform them about the purposes and procedures of the study. From July, 2005 through January, 2006, a total of 1,518 subjects underwent a self-administered dental questionnaire and clinical oral examination. Information on parity was collected from a self-completed questionnaire administered in 1990 for the JPHC Study Cohort I. The final number of subjects used for the analysis was 1,211, after excluding those with missing data for either the outcome or any explanatory variable. Ethical approval of this study was granted by the Ethics Committee of the National Cancer Center in Tokyo and the Tokyo Medical and Dental University Ethical Committee, Japan.

### Parity or number of children

Female subjects were asked their number of childbirths, and male subjects were asked their number of children. The parity or number of children was then divided into five categories: 0, 1, 2, 3, and 4 or more.

### Health behaviors

A self-completed dental questionnaire administered in 2005 inquired about health behaviors such as intake of sweet snacks or drinks (rarely, sometimes, and everyday), presence of a family dentist (yes or no) and smoking status (non-smoker, past smoker, and current smoker).

### Dentition status

Standardized clinical oral examinations of the dentition (excluding third molars) were performed in 2005 by 43 participating dentists using the World Health Organization guidelines [11]. Training and calibration for the dentists were implemented by oral explanation and distributing a handbook describing the detailed clinical criteria prior to the examination. The examination included the number of teeth present, decayed teeth (DT) and filled teeth (FT).

Following the examinations, n-FTUs (Functional Tooth Units of natural teeth), defined as the sum of pairs of opposing natural teeth (i.e., sound, restored and carious teeth), was calculated. Carious teeth with extensive coronal destruction and missing teeth were regarded as non-functional. Because two opposing premolars were defined as one FTU and two opposing molars as two FTUs, a person with a complete occluding dentition had 12 FTUs.

Oral hygiene of teeth or dentures was evaluated by visually examining all teeth or the dentures and was scored as: 1) good = plaque covering less than one-third of tooth surfaces; 2) fair = plaque covering more than one-third but less than two-thirds of tooth surfaces; and 3) poor = plaque covering more than two-thirds of tooth surfaces. The worst score was recorded as representative for the person.

### Statistical analysis

The linear trend of parity or number of children (0, 1, 2, 3, and 4 or more) with demographics, health behaviors and oral hygiene was analyzed by a linear regression model for continuous data and by the Mantel-Haenzel’s chi-square test for categorical data. The adjusted mean of clinical dental outcome variables (numbers of teeth present, decayed teeth, filled teeth and n-FTUs) and linear trend by parity or number of children were assessed using a generalized linear regression model. The analysis was performed both unadjusted-for and adjusted-for age (continuous), education level (low, middle, and high), intake of sweet snacks and drinks (rarely, sometimes, and everyday), presence of a family dentist (yes or no), smoking (non-smoker, past smoker, and current smoker), and oral hygiene of teeth or dentures (good, fair, and poor). All analyses were conducted using SAS 9.0 software (SAS Institute Japan, Tokyo, Japan).

## Results

The numbers of subjects according to the parity 0, 1, 2, 3, and 4 or more among females were 36 (5.5%), 68 (10.5%), 371 (57.2%), 150 (23.1%) and 24 (3.7%), and those by the number of children among males were 25 (4.5%), 53 (9.4%), 336 (59.8%), 130 (23.1%) and 18 (3.2%), respectively (Table 1). Mean age, education level, intake of sweet snacks or drinks, and presence of a family dentist were not significantly associated with parity or number of children. The proportion of female smokers was very low, i.e., less than 10%, and more female smokers were observed with decreasing parity (p for trend = 0.015). Male subjects did not show a significant relationship. The number of children among male subjects was significantly associated with oral hygiene. Subjects with poorer oral hygiene had a higher number of children (p for trend = 0.004). However, no significant relationship was detected among female subjects for parity and oral hygiene.

**Table 1:** Socio-demographics, health behaviors and oral hygiene among the study subjects according to gender and parity or number of children

	**Female**						**Male**					**P for trend**
	**Parity**					**P for trend**	**Number of children**					
	**0**	**1**	**2**	**3**	**≧4**		**0**	**1**	**2**	**3**	**≧4**	
	**(n=36)**	**(n=68)**	**(n=371)**	**(n=150)**	**(n=24)**		**(n=25)**	**(n=53)**	**(n=336)**	**(n=130)**	**(n=18)**	
Age, mean(SD)	64.0(5.21)	65.4(6.01)	66.0(5.72)	65.1(5.84)	66.4(4.83)	0.204	65.5(6.47)	65.7(5.74)	65.0(5.70)	65.7(5.87)	67.2(5.18)	0.280
Education, n(%)												
Low	11(30.6)	22(32.4)	132(35.6)	53(35.3)	13(54.2)	0.074	9(36.0)	16(30.2)	103(30.7)	36(27.2)	7(38.9)	0.939
Middle	18(50.0)	32(47.1)	183(49.3)	78(52.0)	8(33.3)		10(40.0)	31(58.5)	160(47.6)	78(60.0)	5(27.8)	
High	7(19.4)	14(20.6)	56(15.1)	19(12.7)	3(12.5)		6(24.0)	6(11.3)	73(21.7)	16(12.3)	6(33.3)	
Sweet snacks, n(%)												
Rarely	2(5.6)	4(5.9)	23(6.2)	10(6.7)	3(12.5)	0.315	9(36.0)	13(24.5)	59(17.6)	22(16.9)	1(5.6)	0.095
Sometimes	21(58.3)	42(61.8)	199(53.6)	67(44.7)	13(54.2)		13(52.0)	31(58.5)	211(62.8)	85(65.4)	15(83.3)	
Everyday	13(36.1)	22(32.4)	149(40.2)	73(48.7)	8(33.3)		3(12.0)	9(17.0)	66(19.6)	23(17.7)	2(11.1)	
Sweet drinks, n(%)												
Rarely	19(52.8)	30(44.1)	177(47.7)	70(46.7)	12(50.0)	0.543	9(36.0)	18(34.0)	103(30.7)	45(34.6)	6(33.3)	0.971
Sometimes	10(27.8)	18(26.5)	127(34.2)	57(38.0)	7(29.2)		12(48.0)	24(45.3)	155(46.1)	58(44.6)	9(50.0)	
Everyday	7(19.4)	20(29.4)	67(18.1)	23(15.3)	5(20.8)		4(16.0)	11(20.8)	78(23.2)	27(20.8)	3(16.7)	
Family dentist, n(%)												
Yes	33(91.7)	56(82.4)	340(91.6)	136(90.7)	22(91.7)	0.382	20(80.0)	49(92.5)	284(84.5)	113(86.9)	18(100.0)	0.394
No	3(8.3)	12(17.6)	31(8.4)	14(9.3)	2(8.3)		5(20.0)	4(7.5)	52(15.5)	17(13.1)	0(0.0)	
Smoking, n(%)												
Non-smoker	33(91.7)	66(97.1)	361(97.3)	148(98.7)	24(100.0)	0.015	9(36.0)	22(41.5)	120(35.7)	47(36.2)	6(33.3)	0.385
Past smoker	0(0.0)	2(2.9)	6(1.6)	1(0.7)	0(0.0)		13(52.0)	18(34.0)	134(39.9)	52(40.0)	6(33.3)	
Current smoker	3(8.3)	0(0.0)	4(1.1)	1(0.7)	0(0.0)		3(12.0)	13(24.5)	82(24.4)	31(23.8)	6(33.3)	
Oral hygiene, n(%)												
Good	8(22.2)	11(16.2)	62(16.7)	18(12.0)	3(12.5)	0.102	7(28.0)	7(13.2)	36(10.7)	17(13.1)	1(5.6)	0.004
Fair	23(63.9)	47(69.1)	248(66.9)	102(68.0)	17(70.8)		14(56.0)	38(71.7)	217(64.6)	76(58.5)	8(44.4)	
Poor	5(13.9)	10(14.7)	61(16.4)	30(20.0)	4(16.7)		4(16.0)	8(15.1)	83(24.7)	37(28.5)	9(50.0)	

In the bivariate analysis without adjustment for socio-demographic and oral health related variables, there were significant linear trends in some dentition status variables by parity or number of children (Table 2). The number of teeth present significantly declined with the rise of parity in female subjects (p for trend = 0.016), but no corresponding significant relationship was found in males. No significant trends by parity or number of children were observed in the numbers of DT or FT. Regarding posterior occlusion, n-FTUs significantly declined with the rise of parity in female subjects (p for trend = 0.009), similarly in male subjects the number of n-FTUs significantly decreased as the number of children went up (p for trend = 0.035).

**Table 2:** Dentition status (mean±SD) according to gender and parity or number of children

	**Female**						**Male**					**P for trend**
	**Parity**					**P for trend**	**Number of children**					
	**0**	**1**	**2**	**3**	**≧4**		**0**	**1**	**2**	**3**	**≧4**	
	**(n=36)**	**(n=68)**	**(n=371)**	**(n=150)**	**(n=24)**		**(n=25)**	**(n=53)**	**(n=336)**	**(n=130)**	**(n=18)**	
Teeth present												
Crude	18.61(8.97)	19.12(8.62)	18.44(8.51)	16.09(9.24)	14.38(8.74)	0.016	21.80(7.50)	19.04(9.68)	19.41(8.29)	19.79(8.03)	16.83(8.57)	0.086
Adjusted	18.57(7.97)	19.14(7.96)	18.25(7.92)	16.37(7.94)	15.60(7.93)	0.046	20.31(7.73)	18.86(7.70)	19.51(7.68)	19.72(7.67)	18.00(7.72)	0.452
Decayed teeth												
Crude	1.14(2.58)	0.69(1.40)	1.05(2.14)	1.09(1.96)	0.75(2.29)	0.735	1.44(2.97)	1.32(2.62)	1.29(2.48)	1.29(2.43)	1.00(1.41)	0.567
Adjusted	1.23(1.92)	0.58(1.91)	1.07(1.90)	1.05(1.91)	0.83(1.90)	0.750	1.55(2.35)	1.52(2.34)	1.26(2.33)	1.27(2.33)	0.90(2.34)	0.306
Filled teeth												
Crude	9.53(6.05)	11.63(6.80)	11.40(6.19)	10.03(6.72)	9.58(6.26))	0.668	10.52(6.38)	8.81(5.68)	8.61(5.69)	9.65(6.55)	8.94(5.05)	0.540
Adjusted	9.48(6.01)	11.89(6.02)	11.25(5.97)	10.18(5.99)	10.25(5.99)	0.962	10.48(5.71)	8.68(5.68)	8.65(5.66)	9.62(5.67)	8.82(5.70)	0.517
n-FTUs												
Crude	4.94(4.50)	5.00(4.50)	4.54(4.23)	3.75(4.04)	2.58(3.18)	0.009	6.52(4.62)	5.68(4.64)	5.30(4.49)	5.21(4.39)	3.72(4.55)	0.035
Adjusted	4.94(3.88)	4.93(3.87)	4.47(3.85)	3.90(3.86)	3.10(3.86)	0.026	5.76(4.21)	5.61(4.19)	5.33(4.18)	5.22(4.18)	4.39(4.20)	0.249

Adjusted for age, education level, sweet drinks, sweet snacks, presence of family dentist, smoking status, and oral hygiene status.

The multivariate analysis with adjustment for socio-demographic and oral health related variables showed that the numbers of teeth present (p for trend = 0.046) and n-FTUs (p for trend = 0.026) were significantly related with parity in female subjects. The values of these variables had a significantly decreasing trend with increasing parity. No significant associations were observed in male subjects.

## Discussion

This report revealed that parity in Japanese women was related to the dentition status, regardless of socio-demographic and health behavioral factors. Such relationships were not observed in men after adjustment. Association between number of children and oral hygiene or n-FTUs found in bivariate analysis might be intermediated by the socioeconomic factors. Current results imply that higher-parity women are more likely to lose teeth than lower-parity women, probably due to periodontal disease as well as dental caries, which are two major causes for tooth extraction in Japanese adults [12]. Women with four or more children lose nearly three more teeth than women with no or one child.

Several biological mechanisms to explain the lower number of teeth present with parity have been proposed. Pregnancy has a detrimental impact on oral tissues because various biological alterations occur in the oral cavity during the prenatal period. Fluctuations of pregnancy hormones, such as progesterone and estrogen, increase the vascular permeability in the oral cavity and decrease host immunity, increasing the susceptibility of pregnant women to oral infections [13,14]. Further, in pregnant women subgingival periodontal pathogens present a more pathogenic profile, and significant differences in bacterial proportions are found for *Aggregatibacter actinomycetemcomitans*, *Porphyromonas gingivalis*, *Prevotella intermedia/nigrescens*, *Tannerella forsythia*, *Parvimonas micra*, *Campylobacter rectus* and *Fusobacterium nucleatum*[15]. Hormonal variations, as well as changes in the oral flora, place pregnant women at a higher risk for exacerbation of gingivitis and periodontitis [16,17]. The inflammation of periodontal tissues that occurs during pregnancy is temporary and abates after childbirth, but destruction of periodontal tissue persists even after childbirth. Thus, repeated occurrences of periodontal inflammation worsen the existing periodontal diseases [18], and progressive periodontal diseases eventually can result in tooth loss.

Previous studies demonstrated that parity was related to untreated dental caries in the United States [6], and the prevalence of dental caries was higher in pregnant women than in non-pregnant women in Vietnam [19]. Proposed biological mechanisms for an increased susceptibility to caries during pregnancy include changes in saliva and oral flora [20], and changes in immunosuppression [21]. These may contribute to a gender disparity in caries rates [19,22,23]. However, no significant relationship between parity and decayed teeth was detected in this study. This may be because so few decayed teeth were present in our sample. Fewer decayed teeth also reflect a higher number of filled teeth in the present subjects. Universal dental coverage by the national insurance system in Japan may contribute to this result [24]. Studies performed in Africa reported no relationship between parity and dental caries [7,8]. This is mainly because the dental-related environment in Africa is totally different from that in developed countries.

Another plausible reason for aggravation of dental diseases in females, other than biological alterations, is that women with many children may have various reasons for having difficulties getting treatment. Pregnancy and maternity may alter dental utilization patterns. Similarly, dentists’ attitudes and behavior may change when treating pregnant women. Former studies indicate that about half of the pregnant women with dental problems sought no dental care [25], or postponed dental treatment until after childbirth [26]. Therefore, pregnant women with higher risk of dental disease are less likely to receive treatment. Pregnant women may also mistakenly believe that dental problems are a usual and unavoidable experience during pregnancy [27,28]. The popular notion that the fetus takes calcium from the teeth of the mother and that dental treatments can harm the fetus still widely exist, although there is no evidence supporting them [29,30]. In addition, many pregnant women do not think of gingival bleeding as a sign of inflammation, or as a problem that needs dental care [31]. Dentists, generally, are not willing to treat pregnant women and may postpone dental treatments until after childbirth. However it has been confirmed that providing dental treatments in pregnancy, including prophylaxis, restorations, extractions, and periodontal management is generally safe and effective [28,32].

In this study, lower number of n-FTUs was found in higher-parity women. This finding indicates that loss of teeth affects posterior occluding relationships, especially occlusion with natural teeth. The number of FTUs is closely related with masticatory performance [33,34]. Therefore, higher-parity women may not have satisfactory biting and chewing.

One limitation in the study is that intra- or inter-examiner reliability assessments were not carried out. Another limitation is that the subjects had voluntarily participated in the examination, and therefore, may not be a representative sample. Nonetheless, the present study demonstrates that Japanese women with high parity have a higher risk for dental diseases and are more likely to lose teeth, especially posterior occluding relations, compared to those with low parity.

## Conclusion

Parity in Japanese women was related to the dentition status. There are pathological as well as socio-behavioral reasons why parity is related with such dental problems. Therefore, further efforts are needed to narrow the discrepancy in parity-related oral health. To reduce the gap associated with parity, and to promote better oral health in women, it will be necessary to deliver appropriate information and messages to pregnant women and also enlighten oral health professionals about dental management of pregnant women.

## Competing interests

The authors declare that they have no competing interests.

## Authors’ contributions

MU: conceived of the study and conducted statistical analyses and manuscript writing. SO: made contributions to conception and design of the study. MI and ST: contributed to interpretation of data and helped to draft the manuscript. YK: involved in writing the manuscript critically for important intellectual content. All authors read and approved the final manuscript.

## Pre-publication history

The pre-publication history for this paper can be accessed here:

http://www.biomedcentral.com/1471-2458/13/993/prepub
